# Auditory Perceptual Learning in Adults with and without Age-Related Hearing Loss

**DOI:** 10.3389/fpsyg.2015.02066

**Published:** 2016-02-03

**Authors:** Hanin Karawani, Tali Bitan, Joseph Attias, Karen Banai

**Affiliations:** ^1^The Department of Communication Sciences and Disorders, Faculty of Social Welfare and Health Sciences, University of HaifaHaifa, Israel; ^2^The Department of Psychology, Faculty of Social Sciences, University of HaifaHaifa, Israel

**Keywords:** presbycusis, age-related hearing loss, auditory training, speech in noise, time-compressed speech, perceptual learning

## Abstract

**Introduction :** Speech recognition in adverse listening conditions becomes more difficult as we age, particularly for individuals with age-related hearing loss (ARHL). Whether these difficulties can be eased with training remains debated, because it is not clear whether the outcomes are sufficiently general to be of use outside of the training context. The aim of the current study was to compare training-induced learning and generalization between normal-hearing older adults and those with ARHL.

**Methods :** Fifty-six listeners (60–72 y/o), 35 participants with ARHL, and 21 normal hearing adults participated in the study. The study design was a cross over design with three groups (immediate-training, delayed-training, and no-training group). Trained participants received 13 sessions of home-based auditory training over the course of 4 weeks. Three adverse listening conditions were targeted: (1) Speech-in-noise, (2) time compressed speech, and (3) competing speakers, and the outcomes of training were compared between normal and ARHL groups. Pre- and post-test sessions were completed by all participants. Outcome measures included tests on all of the trained conditions as well as on a series of untrained conditions designed to assess the transfer of learning to other speech and non-speech conditions.

**Results :** Significant improvements on all trained conditions were observed in both ARHL and normal-hearing groups over the course of training. Normal hearing participants learned more than participants with ARHL in the speech-in-noise condition, but showed similar patterns of learning in the other conditions. Greater pre- to post-test changes were observed in trained than in untrained listeners on all trained conditions. In addition, the ability of trained listeners from the ARHL group to discriminate minimally different pseudowords in noise also improved with training.

**Conclusions :** ARHL did not preclude auditory perceptual learning but there was little generalization to untrained conditions. We suggest that most training-related changes occurred at higher level task-specific cognitive processes in both groups. However, these were enhanced by high quality perceptual representations in the normal-hearing group. In contrast, some training-related changes have also occurred at the level of phonemic representations in the ARHL group, consistent with an interaction between bottom-up and top-down processes.

## Introduction

Speech perception and communication in noisy environments become more difficult as we age. Specifically, older adults often experience considerable difficulties when listening to speech in the presence of background noise, to competing speech signals or to rapid speech (Pichora-Fuller et al., 1995). Because these conditions are present in everyday situations, many older-adults find it difficult to understand speech in everyday life. These difficulties are often exacerbated by age-related hearing loss (ARHL; Fitzgibbons and Gordon-Salant, 2010) which is one of the most prevalent chronic health conditions among the elderly (Yueh et al., 2003). ARHL is estimated to affect more than 25% of the population aged 60 or more and its incidence is expected to increase with the aging of the population (Roth et al., 2011). While it has been shown that ARHL is the major cause of these speech perception difficulties, research has shown that cognitive functions such as memory and attention also affect these difficulties (Pichora-Fuller, 2008; Humes and Dubno, 2010).

Individuals with sensorineural hearing loss can regain some lost auditory function with the help of hearing aids (Gil and Iorio, 2010; Lavie et al., 2014, 2015), however this is often insufficient when speech perception under non-optimal conditions is considered (Kochkin, 2000; Gordon-Salant, 2005). Therefore, attempts are being made to supplement the rehabilitation process with patient-centered education, counseling, and auditory training, which were hypothesized to help listeners compensate for degradation in the auditory signal and improve communication (Sweetow and Sabes, 2006). In this vein a number of studies have suggested that auditory training may be beneficial for individuals with ARHL (Sweetow and Palmer, 2005; Stecker et al., 2006; Sweetow and Sabes, 2006, 2007; Sweetow and Henderson Sabes, 2010; Lavie et al., 2013). Studies with older adults have shown that even participants with normal pure-tone and speech perception thresholds often report that listening in everyday life has become effortful (Schneider et al., 2002). Thus, the current study specifically asks whether a home-based auditory training approach that mimics the challenges of real-world listening can improve speech perception in normal-hearing and in hearing impaired older adults, and whether the patterns of learning and generalization are influenced by the presence of a hearing impairment.

### Speech processing in younger and older adults

Speech processing involves not only the perception and identification of individual speech sounds and words, but also the integration of successively heard words, phrases, and sentences to achieve a coherent and accurate representation of the meaning of the message being communicated. In this process distinct (but interactive) neural networks process both the acoustic structure and the meaning of speech. The end result, mapping sounds to meaning, relies on matching the output from acoustic and phonetic analyses with stored lexical representations (Davis and Johnsrude, 2007; Hickok and Poeppel, 2007). Thus, accurate speech processing requires the use of voice and emotions cues, the use of silent gaps and duration cues to recognize phonemes, the use of temporal envelope patterns related to the rate of speech and spectral information, and access to and retrieval of semantic information (Price et al., 2005; Pichora-Fuller and Macdonald, 2008). Moreover, cognitive processes such as working memory, selective attention, and the speed at which information can be processed also affect speech understanding (Pichora-Fuller and Singh, 2006). The use of knowledge and semantic context (e.g., phonological and semantic knowledge of phonemes, words, and sentences) is known to enhance recall and comprehension in older and younger adults (Wingfield and Stine-Morrow, 2000; Pichora-Fuller, 2008; Tun et al., 2012).

According to several theoretical accounts, the relative contributions of lower-level sensory and perceptual processes or representations and higher-level cognitive processes (e.g., working memory, semantic processes) to speech recognition may differ between optimal and unfavorable listening conditions [e.g., the Ease of Language Understanding Model (Rönnberg et al., 2013) or the Reverse Hierarchy Theory RHT (Ahissar et al., 2009)]. According to the Ease of Language Understanding Model (ELU), incoming speech is initially processed automatically and a phonological representation of the signal is created. Word recognition (or “lexical access”) should occur if this automatically created representation matches an existing representation in long term memory. However, when an automatically created representation does not match an existing representation in long term memory, for example when the signal is degraded or when sensory processing of the signal is less precise due to hearing loss, an explicit and effortful working memory process is engaged in an attempt to compensate for the mismatch between the phonological representation and long term memory prolonging the recognition process (Rönnberg et al., 2008, 2013). Therefore, under difficult listening conditions or when hearing is impaired listeners are more likely than otherwise to engage in top-down processes that would allow semantic or real-world knowledge to influence speech recognition through working memory or attentional processes (Rönnberg et al., 2013).

Lower level processes are compromised to a greater extent in older-adults with ARHL group than in normal-hearing older adults. For example, older adults with presbycusis required more favorable signal-to-noise ratios (SNRs) to benefit from the ability to predict sentence-final words from sentence context than older adults with normal hearing, even though the magnitude of the context effect was similar in the two groups (Pichora-Fuller et al., 1995). This example also suggests that hearing impairment does not necessarily interfere with the ability to engage top-down processes to support listening. Rather, studies have shown that as supra-threshold auditory processing gradually declines over decades, the brain reorganizes so that more frontal brain areas, including those serving semantic processing and working memory, are activated to a greater extent in older compared to younger brains in conditions in which the performance of older and younger adults is matched (Wingfield and Grossman, 2006; Peelle et al., 2011). As speech becomes less intelligible, processing relies more on top-down influences from frontal areas (Pichora-Fuller et al., 1995; Zekveld et al., 2006). A similar conclusion was reached in an MRI study that found higher correlation between the volume of frontal areas and speech in noise perception in older adults compared to normal-hearing young adults (Wong et al., 2010). Despite this compensatory engagement of higher-level brain areas, older adults experience disproportionate difficulties in understanding speech in ecological conditions that include suboptimal noise conditions and fast talkers. Therefore, successful auditory training in this population should foster an effective balance between bottom-up, signal-based processes, and top-down knowledge-based processes (Pichora-Fuller and Levitt, 2012).

### Auditory training

Auditory training for the purpose of hearing rehabilitation involves active listening to auditory stimuli and aims to improve the ability of participants to comply with the demands of non-optimal listening environments (Boothroyd, 2007; Henderson Sabes and Sweetow, 2007). Home-based auditory training programs were developed to allow adults with hearing loss to engage in perceptual learning, which in turn may lead to better speech understanding and improved communication ability (Sweetow and Sabes, 2007). The consequences of training specific auditory skills are often specific to the trained stimuli (e.g., Wright et al., 1997; Cainer et al., 2008). In addition training outcomes also depend on the trained task (Amitay et al., 2006), suggesting that plasticity is also mediated by cognitive task-specific mechanisms rather than by only the sensory attributes of the trained stimuli. Other factors such as feedback (Amitay et al., 2010) and motivation (Amitay et al., 2010; Levitt et al., 2011; Ferguson and Henshaw, 2015a) likewise influence training outcomes.

Two aspects of learning were typically quantified to document the effects of training on listening skills in the context of hearing rehabilitation—“on-task” learning defined as improvements on the trained tasks and “generalization” defined as improvements in tasks that are not trained directly. On-task learning following auditory training in older adults with ARHL is usually robust, however generalization of learning to untrained tasks or stimuli that were not experienced directly during training does not always occur, or is very small (see Henshaw and Ferguson, 2013 for a similar use of the terms). Robust effects of “on-task learning” were previously reported for syllables and words in older adults with hearing loss (Burk et al., 2006; Stecker et al., 2006; Burk and Humes, 2008; Humes et al., 2009; Ferguson et al., 2014). Burk and colleagues examined the effect of word-based auditory training and focused on word-recognition abilities within a background noise with varied words and talkers. Such training on perceptual distinctions assumes that by resolving lower level sensory issues through training, listening and communication should improve in a bottom-up manner. In their studies, improvements on the trained task were maintained over an extended period of time; however, generalization to untrained words did not occur (Burk et al., 2006). Although there is evidence to suggest that training using multiple talkers promotes greater word-in-noise learning that generalizes to unfamiliar speakers (Burk et al., 2006), such training yields learning that is specific to the content of the trained stimuli and does not always generalize to unfamiliar words, nor familiar words embedded in unfamiliar sentences (Humes et al., 2009).

Other studies suggest that training in ecological tasks, with whole sentences which emphasize top-down processes (such as generating semantic expectations, requiring working memory, and selective attention) might result in wider generalization than training that emphasizes specific auditory capacities (Sweetow and Sabes, 2006; Smith et al., 2009; Anderson et al., 2013a,b). Two home-based training programs were used in previous studies (1) Brain Fitness™ (Smith et al., 2009) that consists of modules designed to increase the speed and accuracy of auditory processing and (2) “listening and communication enhancement” LACE™ (Sweetow and Sabes, 2006) that provides a variety of interactive and adaptive tasks in three categories: degraded speech, cognitive skills, and communication strategies. In the latter program, listeners train on speech recognition in passages on a wide variety of topics, in conditions such as competing speakers, time-compressed speech and speech-in-noise, that mimics the challenges of real-world listening. The overall goal of such ecological training approaches is to improve sensory function, and engage higher level processes that support sensory processing (Schneider and Pichora-Fuller, 2000).

Previous studies evaluated the effects of home-based ecological training on participants with ARHL (Sweetow and Sabes, 2006; Anderson et al., 2013a) and normal-hearing (Anderson et al., 2013b). They found that training changed the neural processing of speech sounds and promoted cognitive and perceptual skills. In one of these studies (Anderson et al., 2013a), participants improved in both physiological (brainstem timing) as well as perceptual assessments (speech-in-noise perception, short-term memory and speech processing) following 40 sessions of computerized home-based auditory training. In another study Anderson et al. (2013b) compared learning in the ARHL group to normal-hearing adults, and found significant training-induced changes in speech-in-noise perception specific to the hearing impaired trained group, with no corresponding changes in the normal-hearing group. Sweetow and Sabes (2006) tested older adult hearing-aid users on trained and untrained measures of speech-in-noise. They reported significant on-task learning effects but only small effects of generalization and only in one of the two untrained tasks with sentences stimuli.

In the current study we trained listeners on speech perception tasks similar to the ecological training programs used in previous studies (e.g., Sweetow and Sabes, 2006; Song et al., 2012). Passages on a wide array of topics were presented in degraded form (noise or time-compression) or in parallel to a competing talker. Listeners had to answer content-related questions and the level of acoustic difficulty was adapted based on their responses. We chose this approach because evidence from normal-hearing individuals and few auditory rehabilitation studies shows that emphasizing top-down processes (selective attention, working memory, use of linguistic, and world knowledge) during training is more effective in terms of generalization than training on basic acoustic features (Borg, 2000; Sweetow and Sabes, 2006; Moore, 2007). Whole sentences are expected to provide top-down lexical feedback in the perceptual learning process (Davis et al., 2005). Thus, the listener may learn to use their stored semantic knowledge about the topic and about language, as well as visually presented verbal information, to facilitate their perception of the “interrupted” acoustic signal. Finally, training on whole sentences is expected to motivate participants and promote compliance with the training regimen.

We focused on adults with mild-to-moderate sensorineural hearing loss who were experiencing hearing difficulties, but had not yet sought intervention for their hearing loss, as well as on normal-hearing adults. To the best of our knowledge, the present study is one of the first studies to conduct home-based training research in everyday listening situations; in fact it is the first of its kind in relation to Hebrew speakers. We expect that training-induced behavioral gains will be observed. Moreover, perceptual learning studies usually ask if learning simple auditory skills can generalize to more complex ones. In the current study, generalization to untrained speech tasks was examined in normal-hearing older adults and those with ARHL. We also ask whether training on complex sounds generalizes to simple acoustic tasks, by testing participants on non-verbal auditory discrimination tasks. The aims of the current study were (1) to examine the efficacy of a home-based auditory training scheme in improving speech perception abilities among normal-hearing older adults and among hearing impaired non-aided older adults. (2) To compare the patterns of training-induced learning between normal-hearing adults and those with ARHL and (3) to assess learning on the trained tasks and transfer to other untrained (speech and non-speech) tasks to study generalization.

## Materials and methods

### Participants

Seventy one adults (44 females) aged 60–71 years (mean age = 66.5 years ± 4 months) with no history of neurological disorders, were recruited for this study. Participants were recruited from the Institute for Audiology and Clinical Neurophysiology at the Interdisciplinary Clinical Center at the University of Haifa, from the Hearing and Speech Center at the Rambam Health Care Campus and through advertisements at the University and Rambam. Recruitment criteria included age 60–72 years, normal-hearing or hearing impairment with no neurologic disorders and Hebrew as a first language. Exclusions from the study were on the basis of audiometric results of asymmetric or conductive hearing loss (*n* = 4), being an existing hearing aid user (*n* = 5), unwillingness to participate in post-test sessions (*n* = 4), inability to control a computer mouse (*n* = 2). Participants provided informed consent and were compensated for their time. All procedures were approved by the Faculty of Social Welfare and Health Sciences, University of Haifa Review Board (approval number 197/12). Pure-tone audiometric thresholds were obtained bilaterally for air conduction at octave frequencies 250–8000 Hz and at 3000 and 6000 Hz and for bone conduction at octave frequencies 250–4000 Hz.

**Figure 1:**
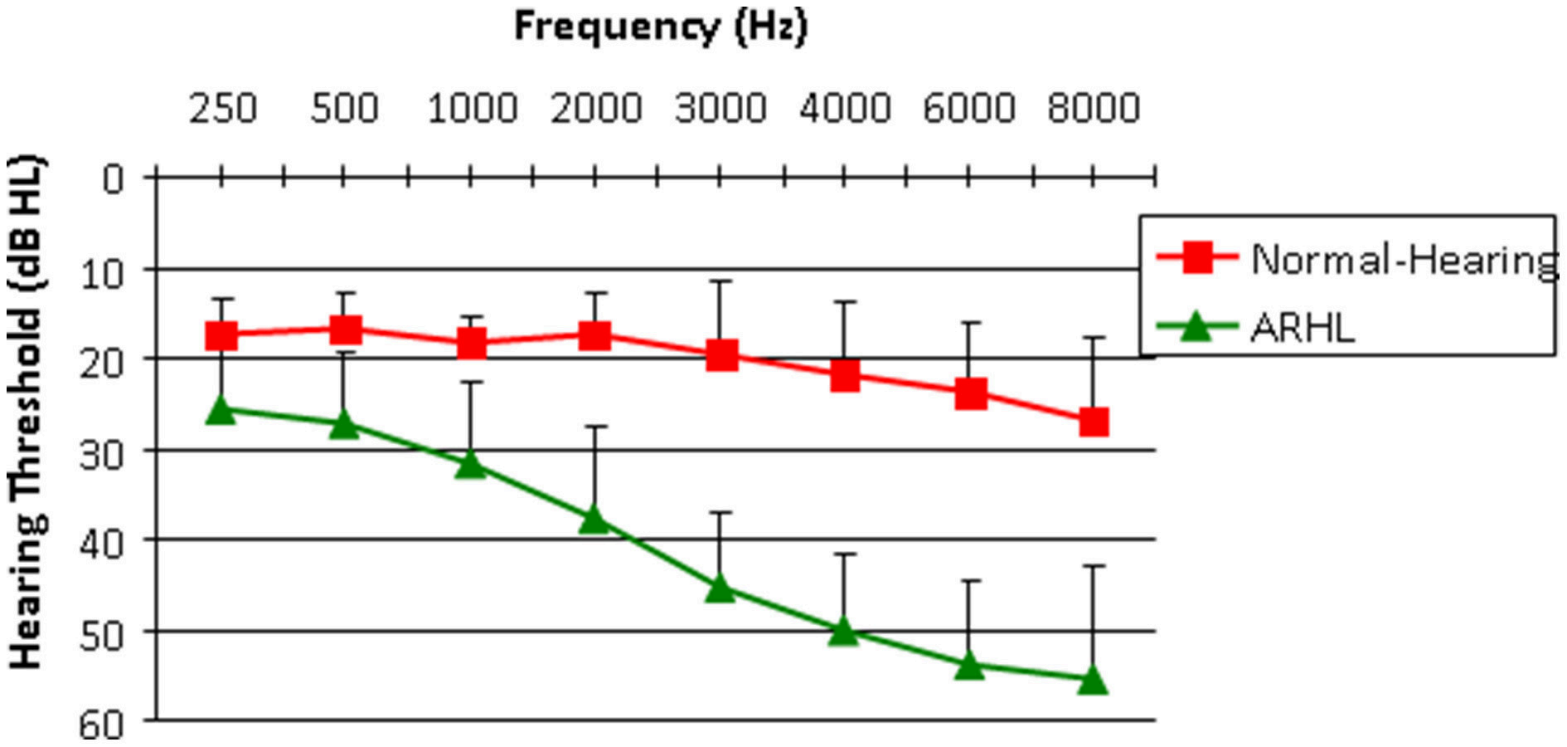
**Audiogram**. Mean air conduction hearing thresholds across ears and participants are plotted for all Normal-Hearing (NH) and Age-Related Hearing Loss (ARHL) participants. Error bars represent standard deviations (SDs).

A total of 56 participants (35 females) met the inclusion criteria reported above and their data is included in the analyses reported in this manuscript. Based on audiometric thresholds participants were divided into normal-hearing (NH, mean age = 64.6 years ± 4.3, *n* = 21) and ARHL (mean age = 67.6 years ± 3.3, *n* = 35) groups; no significant age difference was found between the groups [*t*_(54)_ = 0.7, *p* = 0.59]. The normal-hearing participants had hearing thresholds ≤ 25 dBHL through 6000 Hz and ≤ 30 dBHL through 8000 Hz. Participants with ARHL had symmetrical mild to moderate hearing loss with hearing thresholds ≤60 dBHL through 8000 Hz, and did not use hearing aids either in the past or at the time of the study. Audiograms for both groups are shown in Figure 1. No significant differences between the right and left ears were found in pure-tone average of 500, 1000, and 2000 Hz in air conduction thresholds therefore an average of both ears are shown in Figure 1 [*t*_(110)_ = 0.6, *p* = 0.54]. In addition there were no significant differences in bone conduction thresholds between right and left ears [*t*_(110)_ = 1.03, *p* = 0.305]. All participants received standardized cognitive tests taken from the Wechsler Abbreviated Scale of Intelligence (WASI, Similarities, and Block Design) and the Digit span memory subtest from the Wechsler Intelligence Test (Wechsler, 1997) and showed age normal cognitive function.

### Study design

**Figure 2:**
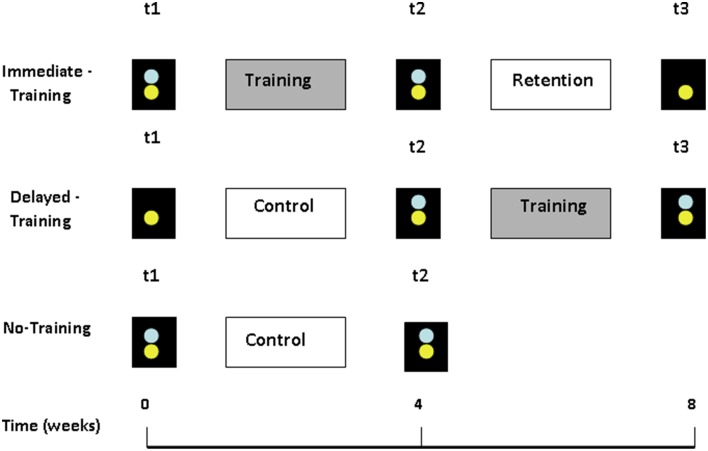
**Study design**. Three testing sessions were conducted for the Immediate-Training and Delayed-Training groups (t1, t2, t3) and two testing sessions for the No-Training group (t1, t2). Immediate-training group underwent training between times t1 and t2 and Delayed-training group between t2 and t3. Blue (top) circles represent testing on trained tasks, yellow (bottom) circles represent testing on untrained tasks.

The study used a randomized, controlled, quasi-crossover design similar in concept to Ferguson et al. (2014). Participants completed three test sessions (see Figure 2). Subgroups of participants underwent auditory training between different test sessions such that overall, participants served as their own untrained controls. All participants (NH and ARHL) underwent a series of tests in session 1 (t1), and then were randomly assigned to either complete the auditory-based training phase immediately (immediate-training, mean age = 65 ± 4.3, *n* = 24; NH = 10, ARHL = 14) or to a waiting phase (delayed-training, mean age = 66 ± 3.1, *n* = 22; NH = 11, ARHL = 11). Another group of participants with ARHL did not train at all (no-training ARHL, mean age = 67 ± 3.4, *n* = 10) and participated in two testing sessions only, see Figure 2. Four weeks after t1 all participants underwent another session (t2). As shown in Figure 2, training occurred between times t1 and t2 for the immediate-training participants and between times t2 and t3 for the delayed-training participants, and the retention period occurred between times t2 and t3 for immediate-training participants. Training data was collected from both training periods (t1–t2, t2–t3); a total of 46 participants underwent the training phase (introduced in the Sections Materials and Methods and Results as trained NH, *n* = 21 and trained ARHL, *n* = 25). Data from the retention period will not be discussed in the current paper.

Details of test sessions for each group: The three testing sessions were conducted at the University of Haifa and included tests on the trained tasks to assess the training effect (on-task learning), and on a series of untrained tasks to assess generalization. As shown in Figure 2, the Immediate-training and No-training groups were tested on the trained and untrained tasks in t1 (pre-test) and in t2 (post-test). For the Immediate-training group, t3 also included tests on the untrained tasks to assess retention (which will not be discussed in the current paper). The delayed-training group was tested only on the untrained tasks in t1, and was then tested on both trained and untrained tasks in t2 and t3.

As shown in Table 1, demographic characteristics and indices of cognitive function (assessed at t1) were similar across all five NH and ARHL groups [*F*_(4, 51)_ ≤ 1.4, *p* ≥ 0.25]. Likewise, demographic and cognitive characteristics were similar across the immediate-training, delayed-training and no-training groups [*F*_(2, 53)_ ≤ 0.92, *p* ≥ 0.86].

**Table 1:** **Means and (SDs) of demographic and cognitive measures across all groups (immediate-training, delayed-training, and no-training) divided into normal-hearing (NH) and Age-related hearing loss (ARHL) groups**.

	**Normal-hearing**		**ARHL**		
	**Immediate-training**	**Delayed-training**	**Immediate-training**	**Delayed-training**	**No-training**
*N*	10	11	14	11	10
Age	64 (4.59)	65 (4.5)	66 (3.08)	69 (2.53)	67.6 (4.42)
Male/female	4/6	8/3	8/6	6/5	7/3
**COGNITIVE FUNCTION**					
Digit span scaled scores	9.5 (1.7)	8.1 (2.08)	9 (2.2)	8 (2.4)	8 (2.6)
Similarities	15.1 (0.8)	14.3 (2.5)	14.3 (2.5)	14.2 (2.9)	14.4 (2.9)
Block design scaled scores	11.1 (1.8)	10.7 (2.3)	10.8 (2.04)	9.3 (2.4)	10.3 (2.1)

### Training protocol and tasks

The trained groups completed 13 sessions of home-based auditory training, each lasting 20–30 min spread over 4 weeks. The training program was designed to improve speech perception in three listening conditions (A) Speech-in-noise (B) Time-compressed speech and (C) Competing speaker. The training tasks were similar in principle to the training procedure introduced in Sweetow and Sabes (2006) and Song et al. (2012). Each session was devoted to one condition, which was practiced for three blocks, except for the last session which included training on all three conditions (one block of each condition). To keep listeners engaged, recordings on a wide variety of topics were used, and in each block a different topic was presented. The auditory training materials were thematic passages of 3–6 min in Hebrew, read by five readers (four male voices and one female) from popular science articles. The passages were broken into content units of 1–2 sentences of about 10 s each, using Audacity software (Audacity, version 1.2. 6). Each unit was followed by a multiple choice question related to the content of the sentences, which was presented visually. Feedback (correct/incorrect response with the correct answer) was also given visually.

During training an adaptive 2-down/1-up staircase procedure was used to adjust the level of difficulty to the performance of each listener based on their individual performance. Improvements with training is reflected by a reduction in the threshold, suggesting that as training progressed listeners could maintain a good level of accuracy even with a more “difficult” (lower quality) stimulus.

The starting values for each day of training were based on the end values of the previous session for each listener in each condition. The speech-in-noise condition sentences were embedded in four-talker babble noise which consisted of two female and two male talkers reading printed prose. The amplitude of each speech signal was maximized to a point just below peak clipping and the four recordings were mixed into a single channel. Various segments of the noise were used to avoid adaptation. The segments were applied pseudo-randomly (i.e., approximately equivalent total number of uses) across sentences to reduce possible effects of amplitude fluctuations that would be present in one noise segment. All noise segments were normalized to an overall root mean square (RMS) level of 70 dB via level 16 (Tice and Carrell, 1997). The adaptive parameter was the signal to noise ratio, where the noise level changed by 1.5 dB. Time-compressed speech adaptive parameter was the compression rate and in the competing speaker's condition, two sentences were presented simultaneously by male and female voices, listeners were instructed to respond to a target speaker and the adaptive parameter was the signal to noise ratio of the two sentences. Mean SNR thresholds of each block was calculated for each participant in speech-in-noise and competing speaker conditions, and mean compression ratio threshold was calculated for each block in the time-compressed speech condition.

The training program was installed by the experimenter (first author HK) on all of the trained participant's personal computers and participants practiced in their homes. Stimuli were presented in sound field via two speakers (Logitech S-0264A, provided by the researchers) placed on either side of the computer and facing the participant (around 45°). The sound level was set at a comfortable listening level, as determined by the trainee, prior to the start of each training session. After the installation of the training program participants completed one practice block for each condition intended to familiarize them with the training program prior to the onset of independent training. At this time participants were also instructed to call the experimenter if they had any questions or if they encountered problems with the program. Subsequently participants were called on a weekly basis to encourage their continued compliance with the training regimen. At the end of training period, the results were uploaded by the experimenter from the personal computers.

Analysis of the training-phase data was conducted on the data of all trained participants (collected between t1 and t2 for the immediate-training group and between t2 and t3 for the delayed-training group). A series of univariate ANOVAs showed no significant differences in the pre-training and post-training results between the normal immediate- and delayed-training groups [*F*_(1, 19)_ ≤ 1.61, *p* ≥ 0.22], and the ARHL immediate- and delayed-training groups [*F*_(1, 23)_ ≤ 1.86, *p* ≥ 0.19], therefore the two groups were combined. A total of 21 NH and 25 ARHL listeners completed training and are referred to as trained listeners or trained groups throughout the Results Sections (Training-Phase Learning, Pre- to Post-Test Learning on the Trained Tasks).

### Pre- and post-training assessments

Pre- and post-training assessments were conducted 4 weeks apart. Data from these sessions was used to assess learning (performance on the trained tasks but with different content) as well as generalization of learning to untrained tasks by comparing changes in performance over time between trained and untrained participants.

#### Learning

Performance on the trained tasks (but with different passages) was used to document learning and determine whether training-related changes were significant in trained in comparison to untrained listeners. For this analysis, data was collected immediately before the first training (pre-test) session and immediately after the final training session (post-test) corresponding to times t1 and t2 for the immediate and no-training groups, and times t2 and t3 for the delayed-training group (see Figure 2, and Section Study Design for more details). Therefore, these analyses, reported in Results—Section Pre- to Post-Test Learning on the Trained Tasks include data from 21 trained NH participants, 25 trained ARHL participants, and 10 untrained ARHL participants. Data on these tasks was not collected for untrained NH listeners, because our main goal was to test the existence of learning changes in the ARHL group. Participants were tested on two blocks of each trained condition in each time point (2 × speech-in-noise, 2 × time-compressed speech and 2 × competing speaker). Differences between pre- and post-tests on the trained tasks were compared between groups.

#### Generalization

Performance on untrained tasks was used to study the transfer of the potential training-induced gains to other speech and non-speech conditions (generalization). These tasks were completed by all subgroups and included (A) a speech-in-noise pseudoword discrimination task, (B) a speech-in-noise sentences task, (C) a duration discrimination task, and (D) a frequency discrimination task.

(A) In the speech-in-noise pseudoword discrimination task participants performed a same/different discrimination task in which 60 pairs of two-syllable pseudowords were presented aurally by a native female speaker with equal numbers of “same” and “different” trials. “Different” trials were minimal pairs (e.g., “same”: /damul/-/damul/, “different”: /malud/-/maluk/), with equal number of pairs from each phonetic contrast and vowel template. The pseudowords were embedded in background four-talker babble noise (same as used in the training paradigm). Pseudowords were used in this test to eliminate the effect of context provided by familiar words, shown to be stronger in individuals with presbycusis compared to near-normal hearing listeners (Pichora-Fuller et al., 1995). (B) Speech-in-noise sentences task, in which listeners were required to make plausibility judgments on 45 Hebrew sentences embedded in the same four-talker babble noise used in the training paradigm. After hearing a sentence listeners had to determine whether the sentence was semantically plausible (“true”) or not (“false”). Both Speech-in-noise tests (pseudowords and sentences) were administered at the most comfortable level for each participant, with a starting SNR value of +5 which was adapted based on their responses with a 2-down/1-up adaptive staircase procedure. The adaptive parameter was the SNR, where the noise level changed by steps of 1.5 dB. All sets of stimuli were RMS-amplitude normalized to 70 dB SPL using Level 16. Just noticeable differences (JNDs) served as the outcome measure for discrimination thresholds in the speech-in-noise pseudowords test, while mean SNR thresholds were used for the speech-in-noise sentences tests. The two speech-in-noise tests (pseudowords and sentences) were used to study generalization to untrained speech-in-noise tasks. (C) Duration discrimination was tested with 1000 Hz reference tones with a standard duration of 200 ms in an oddball procedure. On each trial two identical standard tones and one target tone were presented with an 800-ms inter-stimulus-interval. The duration of the odd tones were adapted based on performance with a 3-down/1-up multiplicative staircase procedure. (D) Frequency discrimination was tested in an oddball procedure with 500 Hz as a reference tone in one task and 2000 Hz reference tone in another task with duration of 500 ms. The frequency difference between the odd and frequent tones was adapted based on performance. The non-speech tasks were administered using a listener friendly interface of 60 trials. These tests were used to determine whether generalization can be observed to untrained basic psychoacoustic non-speech tasks. Each psychoacoustic test lasted ~7–10 min. Visual feedback was provided for both correct and incorrect responses. Stimuli were presented with an initial level of 70 dB SPL, but the tester adjusted the intensity of all speech and non-speech stimuli to a comfortable listening level using the computer's volume setting. Most stimuli were thus presented at the range of 80–83 dB SPL. The level of presentation did not exceed 90 dB SPL.

The generalization tasks were administered to all NH and ARHL participants on times t1 and t2. Thus, these tasks were administered before and after the training period for the immediate-training participants, but before and after the control period for the delayed-training and no-training participants (see Figure 2). Therefore, data from ARHL delayed-training group and the ARHL no-training groups was combined since ANOVA showed no significant differences in the pre-test (t1) and post-test (t2) results [*F*_(1, 19)_ ≤ 4.33, *p* ≥ 0.06]. This resulted in four groups which were compared in subsequent analyses: two groups were tested before and after their training period—the immediate-training NH (*n* = 10) and immediate-training ARHL (*n* = 14) groups and two groups were tested before and after their control period NH (delayed-training, *n* = 11) and ARHL (delayed training + no-training groups, *n* = 21). Data was analyzed using repeated measures ANOVA with two between subject factors (during-training vs. during-control period and NH vs. ARHL) and one within subject factor: time (t1 vs. t2). Shapiro-Wilk tests were used to confirm that the data was normally distributed within each group (*p* > 0.1). In addition, Levene tests confirmed that variances were homogeneous across groups within each analysis (*p* > 0.16).

## Results

### Training-phase learning

Forty-one out of 46 trained participants, from both the NH or ARHL groups, completed all 13 sessions of the auditory training program, showing a high level of compliance with no dropouts; five additional participants completed 10–11 sessions. Data from all 46 trained participants was therefore included in the statistical analysis.

**Figure 3:**
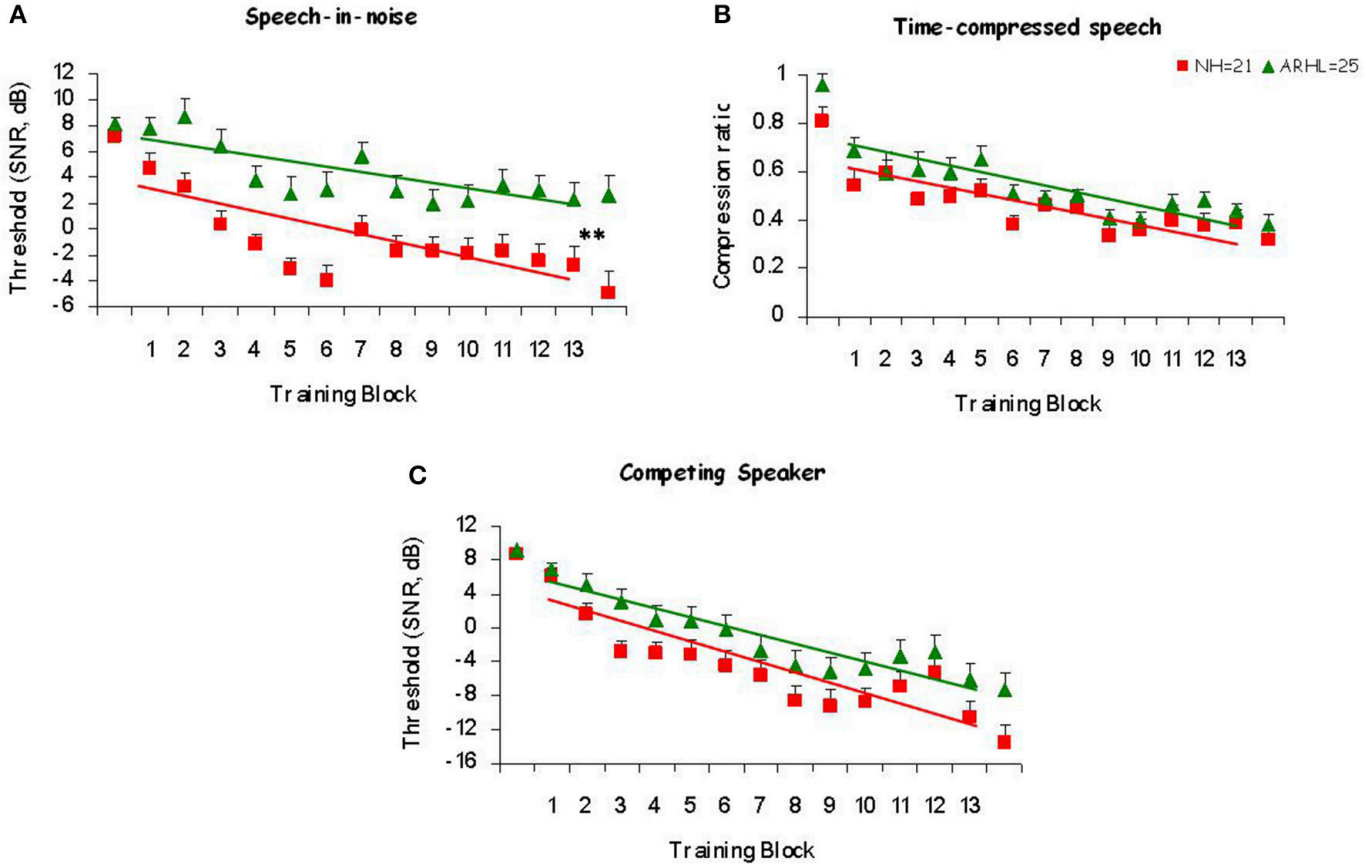
**Learning curves**. Mean thresholds as a function of the trained block for trained Normal-Hearing (NH) and trained Age-Related Hearing Loss (ARHL) participants in **(A)** Speech-in-noise **(B)** Time-compressed speech and **(C)** Competing speaker conditions. Mean signal-to-noise ratio (SNR) thresholds of each block was used as the dependent measure in speech-in-noise and competing speaker conditions and the compression ratio was used for the time-compressed speech condition. Regression lines and slopes of the learning curves **(A)** for trained NH are shown in red and for trained ARHL in green. ^**^p < 0.01.

In order to determine whether participants improved during training, and whether this depended on their hearing status, linear curve estimation was performed on the performance of the group in each training condition across sessions (Figure 3). These analyses (see Table 2 for details) revealed a good fit of the linear curves to the data with significant R-squared values (R-squared > 0.43, *p* < 0.01) that, suggests that a linear improvement across sessions accounts for a significant amount of the variance in performance.

**Table 2:** **Linear curve estimation model of group data**.

		**R-squared**	***F*(1, 11)**	***p***
Speech-in-noise	NH	0.58	15.41	0.002
	ARHL	0.43	8.12	0.009
Time-compressed speech	NH	0.73	22.16	0.000
	ARHL	0.73	24.83	0.000
Competing speaker	NH	0.73	30.48	0.000
	ARHL	0.83	57.10	0.000

*R-squared, F-values with degrees of freedom and p-values are presented across conditions for trained normal-hearing (NH) and trained Age-related hearing loss (ARHL) groups*.

To compare the amount of training-induced changes between groups (NH and ARHL) the linear slopes of the individual learning curves were calculated for each participant in each training condition. As shown in Table 3, mean slopes were significantly negative (*p* < 0.01) in both trained groups and across all three training conditions. In the speech-in-noise condition, learning curves were significantly steeper in the NH than in the ARHL group [*t*_(44)_ = −2.05, *p* = 0.046]. No significant differences were found between the learning-curve slopes of NH and ARHL participants in the time-compressed speech condition [*t*_(44)_ = 0.65, *p* = 0.52] and in the competing speaker condition [*t*_(44)_ = −0.76, *p* = 0.45].

**Table 3:** **Means and (SDs) of the individual linear learning slopes for trained normal-hearing (NH) and trained Age-related hearing loss (ARHL) groups**.

	**NH**	**ARHL**	***t***	***p***	**95% confidence interval of the difference**
Speech-in-noise	−0.59 (0.4)	−0.32 (0.3)	−2.05	0.04	[−0.527, −0.005]
Time-compressed speech	−0.02 (0.01)	−0.02 (0.01)	0.65	0.52	[−0.008, 0.016]
Competing speaker	−1.21 (0.8)	−1.04 (0.7)	−0.76	0.45	[−0.615, 0.277]

*t-values, p-values of the group comparison and 95% confidence interval of the difference between groups are also shown*.

Visual inspection of the data (see Figure 3) suggests that the rate of learning may have changed over the course of training with an initially rapid learning phase followed by a slower learning phase. Therefore, two-line linear curves were also fitted to the group data, separately for sessions 1–6 and 7–13 in each condition (see Supplementary Material). These models showed a good fit in some conditions and groups. Therefore, only for conditions in which both groups showed a significant fit to the model, individual slopes were calculated and the slopes were compared between groups. The results were similar to those obtained with the one-line model (see Supplementary Material for details).

Taken together, these data suggest that training-phase learning was observed in both the normal-hearing and the ARHL trained groups. Both trained groups showed a similar amount of learning over the course of training in the time-compressed speech and competing speaker conditions. However, in the speech-in-noise training condition normal-hearing group showed more improvements than ARHL group.

#### Pre- to post-test learning on the trained tasks

**Figure 4:**
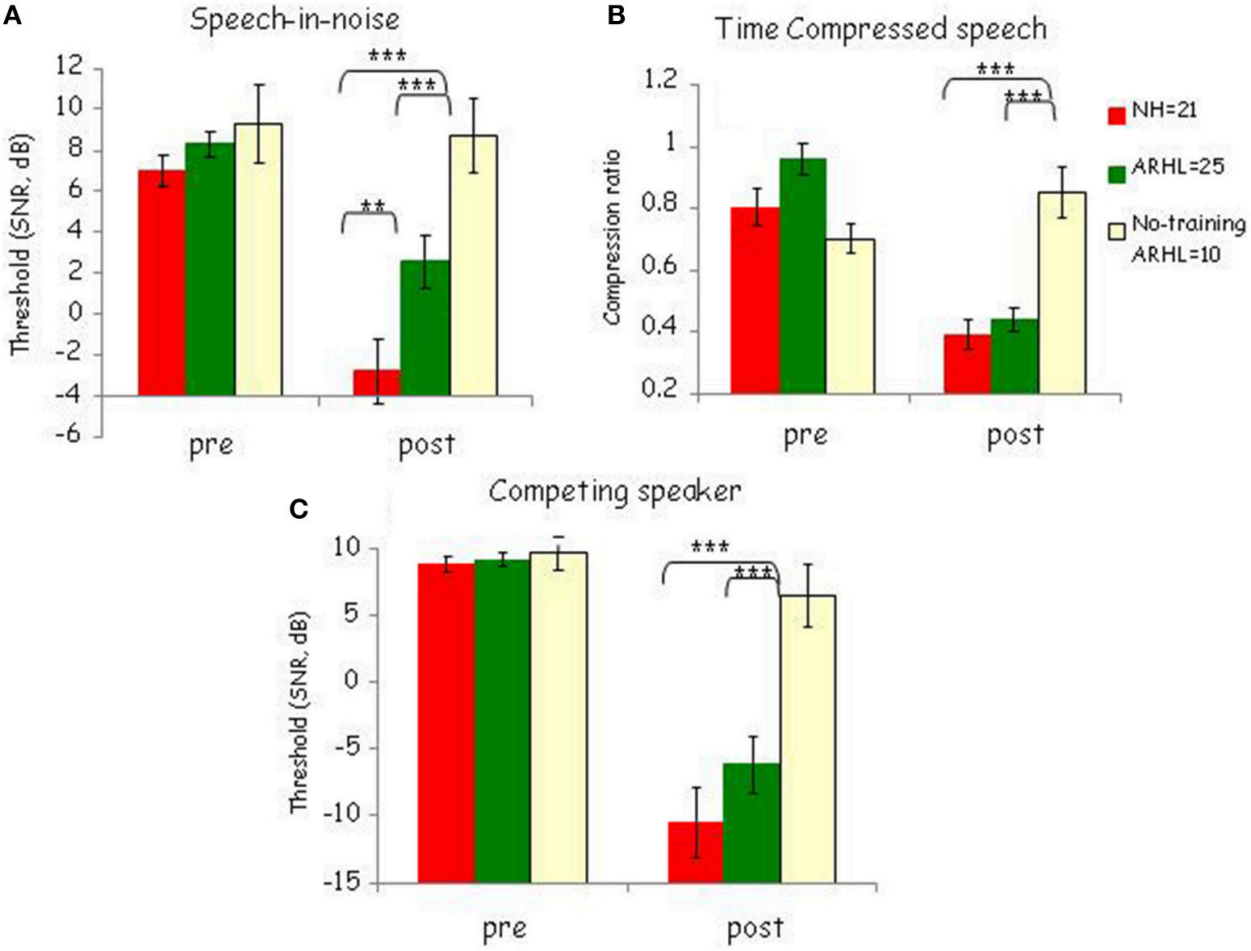
**Pre-to-post learning effects**. Pre- and post-test performance in trained normal-hearing [NH, trained ARHL (ARHL)] and no-training ARHL group for the three conditions: **(A)** Speech-in-noise **(B)** Time-compressed speech and **(C)** competing speaker. Mean signal-to-noise ratio (SNR) thresholds and SDs are shown for the speech-in-noise and competing speaker conditions and mean compression ratio thresholds and SDs are shown for the time-compressed speech condition. ^***^p < 0.001; ^**^p < 0.01.

To determine whether training resulted in greater pre- to post-test changes in trained than in untrained participants and as a function of hearing status, pre- and post-test performance on each of the trained conditions was compared across the three groups (see Figure 4) using a repeated measures ANOVA with group (NH, ARHL, no-training ARHL) as a between-subject factor and time (pre-test, post-test) as a within-subject factor followed by *post-hoc* tests. As explained in Section Learning, the trained tasks was administered immediately before the first training session(pre-test) and immediately after the final training session (post-test) corresponding to times t1 and t2 for the immediate and no-training groups, and times t2 and t3 for the delayed-training group (see Figure 2). Therefore, these analyses include data from 21 trained NH participants, 25 trained ARHL participants, and 10 untrained ARHL participants.

The results showed a statistically significant effect of time and group. Performance on all three trained conditions was significantly influenced by both time [pre vs. post—speech-in-noise: *F*_(1, 53)_ = 32.50, *p* < 0.0001, $\eta_{p}^{2}=$ 0.38; time-compressed speech: *F*_(1, 53)_ = 47.21, *p* < 0.0001, $\eta_{p}^{2}=$ 0.47; competing speakers: *F*_(1, 53)_ = 109.98, *p* < 0.0001, $\eta_{p}^{2}\;=$ 0.68] and group [speech-in-noise: *F*_(2, 53)_ = 7.8, *p* < 0.001, $\eta_{p}^{2}=$ 0.23; time-compressed speech: *F*_(2, 53)_ = 6.01, *p* < 0.001, $\eta_{p}^{2}=$ 0.27; competing speakers: *F*_(2, 53)_ = 9.68, *p* < 0.0001, $\eta_{p}^{2}=$ 0.27]. The time × group interactions were also significant: speech-in-noise: *F*_(2, 53)_ = 9.01, *p* < 0.001, $\eta_{p}^{2}\;=$ 0.26; time-compressed speech *F*_(2, 53)_ = 28.77, *p* < 0.001, $\eta_{p}^{2}=$ 0.52; competing speaker *F*_(2, 53)_ = 14.41, *p* < 0.001, $\eta_{p}^{2}=$ 0.35 (see Figure 4). The significant differences between pre- and post-tests stem from greater changes in both trained groups than in the no-training group. *Post-hoc* Tukey HSD analysis showed significant (*p* < 0.001) pairwise comparisons between the no-training group with each trained group (NH and ARHL) for all three conditions [speech-in-noise: *F*_(2, 53)_ = 10.89, time-compressed speech: *F*_(2, 53)_ = 12.32 competing speaker: *F*_(2, 53)_ = 12.43]. Moreover, *t*-test analyses showed a significant effect of time for both NH and ARHL trained groups on the three conditions [ARHL: speech-in-noise: *t*_(33)_ = −2.96, *p* < 0.001; time-compressed speech *t*_(33)_ = −3.87, *p* < 0.001; competing speaker *t*_(33)_ = −3.57, *p* < 0.001. NH: speech-in-noise: *t*_(29)_ = −4.38, *p* < 0.001; time-compressed speech *t*_(29)_ = −4.22, *p* < 0.001; competing speaker *t*_(29)_ = −4.97, *p* < 0.001]. On the other hand, as can be seen in Figure 4, untrained listeners hardly changed between the two points and no significant differences between pre- and post-tests for the untrained group were found in any condition [speech-in-noise: *t*_(9)_ = 1.03, *p* = 0.57; time-compressed speech: *t*_(9)_ = −2, *p* = 0.95; competing speaker: *t*_(9)_ = 1.8, *p* = 0.1]. Taken together, training induced learning was observed for trained tasks in both normal-hearing and ARHL trained groups in all conditions, untrained listeners did not show any changes between pre- and post-tests and significant differences were observed between trained and untrained listeners; all these confirm that trained listeners improved more than untrained listeners between the pre- and the post-tests. Moreover, normal-hearing trained group significantly outperformed ARHL trained group in the speech-in-noise condition in the post-test session, [Hearing group effect: *F*_(1, 44)_= 7.97, *p* < 0.01, Figure 4A], consistent with the steeper learning curves observed in this group during training.

#### Generalization

**Figure 5:**
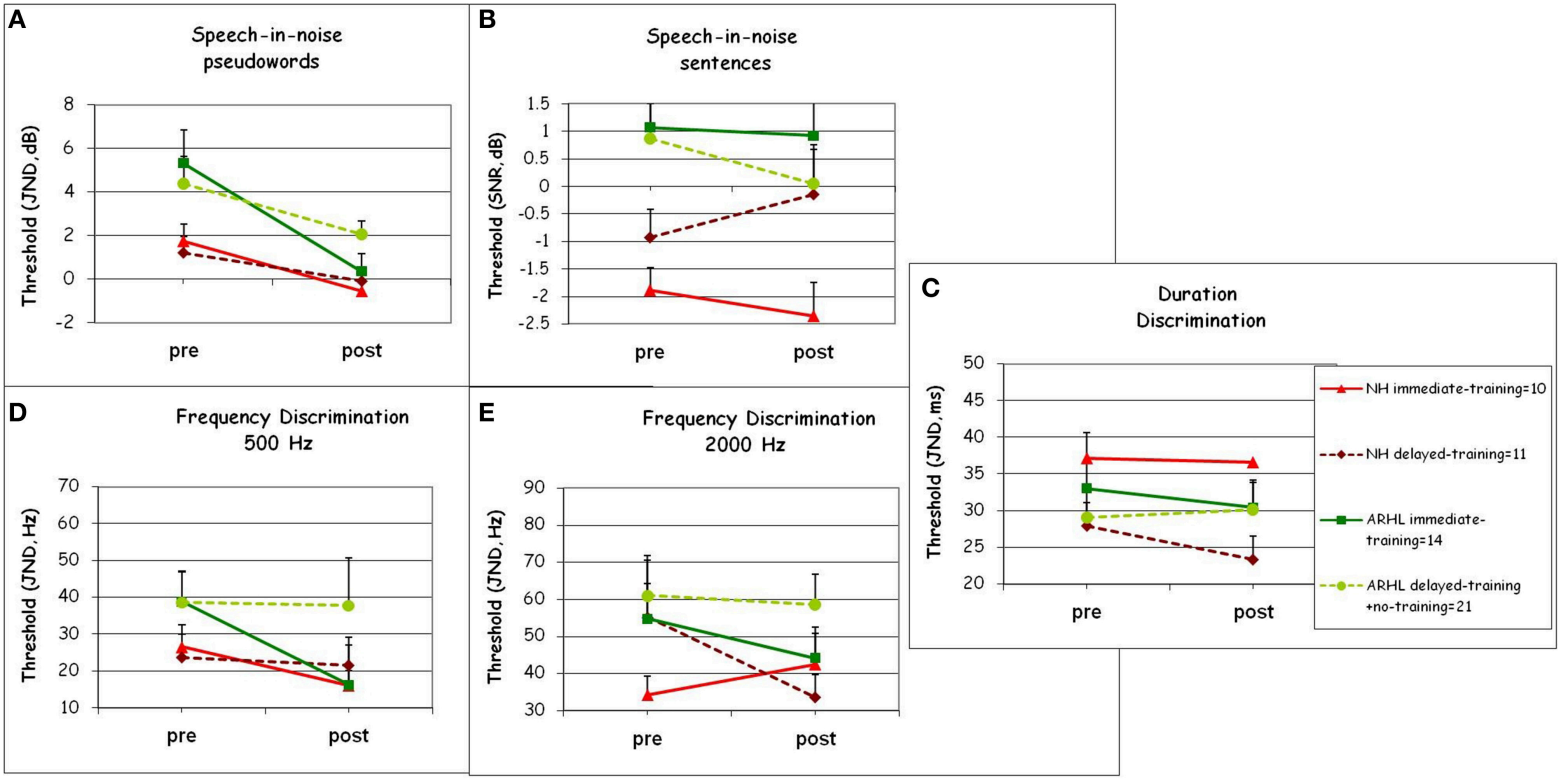
**Generalization**. Means and SDs of **(A)** speech-in-noise pseudowords and **(B)** speech-in-noise sentences thresholds in dBs **(C)** duration discrimination in milliseconds (ms) **(D)** 500 Hz frequency discrimination and **(E)** 2000 Hz frequency discrimination thresholds in Hz, obtained from pre- and post-tests for Normal-Hearing (NH) and Age-Related Hearing Loss (ARHL) groups. For the subgroups: NH immediate-training, ARHL immediate-training, NH delayed-training, and ARHL delayed-training + no-training. See Materials and Methods—Section Generalization for subgroups division.

To study the transfer of learning and to determine whether training resulted in greater pre- to post-test changes during-training than during-control period and as a function of hearing level, pre- (t1) and post-test performance (t2) on the untrained tasks was compared across the immediate-, delayed-, and the no-training groups between the times t1 and t2 (see Figure 2). As shown in the Materials and Methods—Section Generalization. The participants were divided into four groups (1. NH immediate-training, 2. ARHL immediate-training, 3. NH delayed-training, 4. ARHL delayed-training + no-training, see Figure 5) using a repeated measures ANOVA with two between subject factors (training and hearing groups) and one within subject factor, time (pre vs. post). Mean group thresholds are shown in Figure 5, across all untrained tasks, as a function of hearing and training factors, for speech (speech-in-noise pseudowords and sentences, Figures 5A,B) and non-speech tasks (duration discrimination Figure 5C and frequency discrimination, Figures 5D,E).

##### Speech in noise tests

Significant effects of hearing group were found in both speech-in-noise tests (Figures 5A,B), where normal-hearing participants significantly outperformed participants with ARHL [speech-in-noise pseudowords: *F*_(1, 52)_ = 8.14, *p* = 0.006, $\eta_{p}^{2}=$ 0.14; speech in-noise sentences: *F*_(1, 52)_ =11.13, *p* = 0.002, $\eta_{p}^{2}=$ 0.18]. A significant main effect of time was observed only in the speech-in-noise pseudowords task [time: *F*_(1, 52)_ = 23.42, *p* < 0.001, $\eta_{p}^{2}=$ 0.32]. No significant effect of time was shown in the speech-in-noise sentences task. A significant interaction of time × training group was observed only in the speech-in-noise pseudowords task [*F*_(1, 52)_ = 4.47, *p* = 0.036, $\eta_{p}^{2}=$ 0.08]. This interaction stems from a significant effect of time [*F*_(1, 33)_ = 21.01, *p* < 0.001], and a significant interaction of time × training group [time: $\eta_{p}^{2}=$ 0.40; time × train: *F*_(1, 33)_ = 6.24, *p* = 0.018, $\eta_{p}^{2}=$ 0.16] only for the ARHL groups. The interaction time × training was not significant among normal-hearing participants. Therefore, transfer of learning was observed only for the speech-in-noise pseudowords task and only in ARHL group.

##### Duration and frequency discrimination tasks

No significant differences were observed between any of the groups on these tasks (neither hearing differences nor training vs. control period differences, *p* > 0.4). There was no main effect of hearing group (*p* > 0.10) or training group (*p* > 0.12) in any of the non-speech tasks. In frequency discrimination 500 Hz task, there was a main effect of time [*F*_(1, 52)_ = 5.42, *p* = 0.026], but without any interaction with either hearing group (*p* = 0.13) or training group (*p* = 0.89). These results indicate that there was no transfer of learning to the duration discrimination or frequency discrimination tasks in any of the groups (Figures 5C–E).

## Discussion

The present study tested the effect of a home-based training program in everyday listening situations, specifically focused on older adults with mild-to-moderate sensorineural hearing loss who experienced hearing difficulties but did not have hearing aids as well as normal-hearing listeners in the same age range. The outcomes of training on speech perception were compared between normal-hearing adults and those with ARHL. The outcomes of training on generalization to other speech and non-speech tasks were assessed.

The major outcomes of the current study were: (i) Robust training-induced learning effects were found in both normal-hearing and individuals with ARHL, and for the trained tasks these were not limited to the trained materials. (ii) The normal-hearing group showed more learning than the ARHL in the speech-in-noise trained condition. (iii) Generalization to the perception of pseudowords in-noise was observed in the ARHL group only. (iv) The perception of sentences in-noise, duration discrimination and frequency discrimination did not improve in either of the trained groups. Together these findings suggest that although learning remains robust in older adults with normal hearing and in older adults with ARHL, generalization is limited.

### Learning and generalization

#### Learning on the trained tasks

Consistent with previous studies (Sweetow and Sabes, 2006; Humes et al., 2009), and as expected, learning was observed in the trained groups. In the current study, significant training-phase learning was observed in both normal-hearing and ARHL. Participants performed significantly better at the end of the training period than on the initial blocks (Figure 3), indicating that participants' understanding of speech improved over the course of training, in all three conditions: speech-in-noise, time-compressed speech, and competing speaker. Furthermore, between the pre- and post-tests both ARHL and normal-hearing participants improved on the trained conditions more than untrained participants (Figure 4). The normal-hearing and the ARHL groups showed similar patterns of learning over the course of training in the time-compressed speech and competing speaker's conditions as evident by their overlapping learning curves (Figure 3). On the other hand, in the speech-in-noise condition, the learning curves were significantly steeper in the normal-hearing than in the ARHL group (Figure 3), suggesting that on this condition, training had a greater influence on normal-hearing listeners than on listeners with ARHL. The current study is the first to compare between the training outcomes of normal hearing and ARHL groups. These groups are defined by a difference in lower level sensory processes. Even though the training program was designed to emphasize higher level top-down cognitive processes the difference in learning between groups suggests that the poor quality of perceptual representations in ARHL reduced the benefit of this type of training. It is possible that the use of hearing aids might improve the quality of representations and therefore could enhance the benefits of training.

#### Generalization

Although learning on the trained conditions was not stimulus specific (Figure 4), the magnitude of training-induced transfer to other speech-in-noise tasks was small (Figure 5A). Transfer was limited to the pseudowords task and to the ARHL group. This finding is consistent with the findings of Anderson et al. (2013b) where significant improvements in the speech-in-noise outcome measure were specific to the hearing impaired group and generalization was not shown in the normal-hearing group. On the other hand, in the current study, training (in both ARHL and normal-hearing) did not generalize to an untrained speech-in-noise sentence task. In this task, listeners had to judge the semantic plausibility of sentences embedded in noise. This task was different from the trained task, in which listeners were asked multiple-choice questions about the content of the sentence they had heard. So despite using the same babble noise, the change in task requirements was sufficient to preclude generalization. Moreover, no transfer was found in either group to more basic psychophysical abilities such as duration or frequency discrimination (Figures 5C–E). These findings suggest that the type of training used in the current study affected higher level task-specific cognitive processes and did not enhance low-level auditory processing of duration or frequency.

The small effect of generalization observed in the ARHL group was also reported in previous training studies using a similar training program (e.g., Sweetow and Sabes, 2006). Sweetow and Sabes reported only small effects of generalization to speech outcomes in adults with ARHL, and only in one of the two untrained tasks with sentences stimuli. In contrast, normal-hearing young adults showed generalization to untrained speech tasks when trained with the same program, suggesting that training improved the neural representation of cues important for speech perception (Song et al., 2012). Altogether these results suggest that the restricted generalization in the current study, in which both groups were of older age, is associated with the degenerative changes that occur due to aging or hearing loss or both.

One potential interpretation for the discrepancy between learning and generalization in the ARHL group is that during training, although listeners focused on the content of the sentences and not on the acoustic/phonetic characteristics of the stimuli, the low quality of the signal (due to both their auditory loss and noise) had driven listeners to rely on lower-level sensory representations that were not sentence specific. Although the ability to use lower-level sensory representations may have been helpful when making decisions about pseudowords, it would not have been enough when new semantic demands were imposed by semantic judgment task [see Ahissar et al., 2009 for the detailed theoretical framework and (Banai and Lavner, 2014) for a previous discussion in the context of the perceptual learning of speech]. Consistent with this interpretation, it is plausible that mid-level sensory representations were used during training and that was shown in the pseudowords task. Learning did not reach as high as the levels of sentences representations and it did not go as low as the acoustic parameters of frequency or duration. This may be due to the type of task and feedback used during training, or may be a more general feature of auditory training as suggested by small generalization effects observed in previous studies see Henshaw and Ferguson (2013).

An alternative hypothesis is that generalization at the perceptual level of speech in noise could be identified with other outcome measures not used in the current study (Amitay et al., 2014), such as identification of real words or identification of key words in a sentence. Moreover, changes in higher level processes could perhaps be identified with tests of working memory and attention (Ferguson and Henshaw, 2015b). On the other hand, a variety of outcome measures have been used across previous studies, but only small effects of generalization have been reported. Therefore, auditory training may prove useful in hearing rehabilitation, but only if future studies converge on training regimens that yield greater generalization than observed with the regimens studied so far. A potential way forward is to combine the different types of training approaches in order to offer generalization benefits to real world listening abilities as suggested by Ferguson and Henshaw (2015b).

#### Comparisons between normal-hearing and ARHL groups in the generalization of learning

Differences between normal-hearing and ARHL were shown when looking at the transfer tests (Figure 5A); where a significant transfer effect, albeit small, was observed in the ARHL in the pseudowords task, but not in the normal-hearing group. The differences between the normal-hearing and the ARHL groups concerning transfer to the speech-in-noise pseudowords test may be consistent with the processing model introduced in the introduction (Section Speech Processing in Younger and Older Adults). It is plausible that for normal-hearing participants the bottom-up acoustic information was still reliable and sufficient, therefore it matched the lexical representations and there was no need to divert attentional resources to low-level representations during training. In the ARHL group, lower-level and lexical representations did not automatically match, making it necessary to devote attentional resources to the matching process. This additional burden in the ARHL group may have increased the reliance on bottom-up perceptual processes which were generalized to pseudowords.

### Compliance and subjective outcomes

Our training paradigm tried to mimic the challenges of real-world listening and consisted of blocks of sentences in a wide variety of topics. In addition to enhancing reliance on top-down processes the aim of this approach was to enhance motivation and compliance with the training program. It was previously shown that increased time on task is positively associated with gain in understanding speech in noise (Levitt et al., 2011). Thus, the training paradigm in the current study engaged participants resulting in a high rate of compliance (90%) similar to previous reports by Stecker et al. (2006). The improvement on the trained conditions suggests that participants with ARHL can benefit from an improved SNR adjustment to compensate for the inaudibility of high frequencies; such improvements though, are hard to accomplish in many everyday settings. However, despite the lack of evidence concerning transfer of learning in objective measures, more than 50% of the normal-hearing and 75% of the ARHL trained listeners reported that training was helpful in their communication with their grandchildren “*especially those who speak really fast*,” and “*understanding what is being said in noisy environments*” suggesting that training may result in subjective benefits.

## Conclusions

We suggest that most training-related changes in the current study occurred at a higher level of task-specific cognitive processes in both groups, as evident by the lack of generalization to the sentence task, and to the frequency and duration discrimination tasks. Given that the difference between the normal-hearing and ARHL groups is defined based on lower level acoustic and perceptual processing, the larger learning gains in the normal-hearing group suggests an interaction between bottom-up and top-down processes. Namely, learning related changes in high level task-related cognitive processes is enhanced by the high quality of perceptual representations in the normal-hearing group.

Furthermore, the finding of generalization to pseudowords, only in the ARHL group, suggests that some learning related changes have also occurred at the level of identifying phonemic representations in this group. Presumably, because perceptual and phonemic representations were of low quality in the ARHL group, the training program has affected this level of representations in ARHL more than in the normal-hearing group.

Taken together, it was observed in the current study that the auditory training that was used, benefits people with mild-to-moderate hearing loss. It is left for future research to measure top-down processing strategies in order to enhance our understanding of the effects of training. There may be more effective training methods to add to the current training program; perhaps this requires more diverse training—in many more tasks, or more intensive training over a very long period of time or change in the type of feedback used. Finally, studies into the training regimen that yields more generalization are needed.

## Author contributions

HK, TB, JA, and KB designed the study; HK collected and analyzed the data; HK, TB, and KB wrote the manuscript. All authors approved the final version of the manuscript.

### Conflict of interest statement

The authors declare that the research was conducted in the absence of any commercial or financial relationships that could be construed as a potential conflict of interest. The reviewer, Carine Signoret, and handling Editor declared their shared affiliation, and the handling Editor states that the process nevertheless met the standards of a fair and objective review.
