# Supplementary material for: Auditory Perceptual Learning in Adults with and without Age-Related Hearing Loss
Source: Front Psychol. 2016 Feb 3;6:2066. doi: 10.3389/fpsyg.2015.02066 (PMC4737899; doi:10.3389/fpsyg.2015.02066)
Supplement: Supplementary file 1 [file DataSheet1.pdf]

Supplementary materials

A visual inspection of Figure 3 in the manuscript suggests that the rate of learning might change during the course of the training phase, and therefore that a two-line function with a steeper slope over the initial training blocks and a shallower slope over later blocks might provide a better fit of the data. To this end, the training phase data was re-analyzed using a two-line model: Linear regression lines were fit as a function of block number, in two periods of training (1) blocks 1-6 (slope-I), and (2) blocks 7-13 (slope-II), as shown in Figure 1 supplementary.

In order to determine whether participants improved during training, and whether this depended on their hearing status, two-line linear curve estimation was performed on the performance of the group in each training condition in the two periods (Figure 1 supplementary). These analyses revealed a good fit of the linear curves to the data with significant R-squared values ($p < 0.01$) in the following conditions: speech-in-noise condition slope-I, slope II in NH group and slope-I in ARHL and in the competing speaker condition slope-I in NH and ARHL groups as shown in Table 1 supplementary. Therefore comparisons between NH and ARHL slopes were calculated for the speech-in-noise and competing speakers conditions.

Linear slopes of performance over the first six training blocks (slope-I) and the last seven training blocks (slope-II), were calculated for each participant in each training condition and presented in Table 2 supplementary. Individual slopes are calculated only in conditions in which the two-line groups model showed a significant R-squared value. In order to compare the learning curves between groups (NH and ARHL (in the speech-in-noise and competing speaker conditions). No significant differences were found between the learning-curve slopes of NH and ARHL participants in the competing speaker condition slope-I ($t_{(44)} = -1.69$, $p = 0.09$) and in slope-II ($t_{(44)} = -0.004$, $p = 0.99$). In the speech-in-noise condition, learning curves were significantly steeper in the NH than in the ARHL group in slope-I ($t_{(44)} = -2.51$, $p = 0.01$) but not in slope-II ($t_{(44)} = -0.17$, $p = 0.86$).

Taken together, similar to the results shown in the manuscript section 3.1; these data suggest that training-phase learning was observed in both the normal-hearing and the ARHL trained groups. Both trained groups showed a similar amount of learning over the course of training in the time-compressed speech and competing speaker conditions. Moreover a similar finding to the single-line model addressed in the manuscript was also observed; in the speech-in-noise training condition normal-hearing group showed more improvements than ARHL group, however in the two-line model this difference was shown only in the first six blocks. This improvement may have yielded the significant difference between both NH and ARHL groups when tested on the performance of the trained tasks. Since similar main effects were shown for the single-line and two-line models, the single-line model was addressed in the manuscript because it provides a more parsimonious summary of the training-phase learning.

Legend to Table 1 supplementary: Linear curve estimation model of group data. R-squared, F values with degrees of freedom and p values are presented across conditions for trained normal-hearing (NH) and trained Age-related hearing loss (ARHL) groups over the first six training blocks (slope-I: blocks1-6) and the last seven training blocks (slope-II: blocks 7-13).

		Slope-I			Slope-II		
		**R-squared**	**F(1,4)**	**p**	**R-squared**	**F(1,5)**	**p**
**Speech-in-noise**	**NH**	0.99	278.4	0.000	0.76	20.43	0.000
	**ARHL**	0.84	22.75	0.008	0.27	1.88	0.22
**Time-compressed speech**	**NH**	0.60	5.65	0.07	0.25	1.66	0.25
	**ARHL**	0.40	2.68	0.17	0.26	0.38	0.56
**Competing speaker**	**NH**	0.87	17.34	0.009	0.30	0.37	0.56
	**ARHL**	0.59	67.37	0.001	0.30	0.59	0.47

Legend to Table 2 supplementary: Means and (SDs) of the two-line linear model learning slopes for trained normal-hearing (NH) and trained Age-related hearing loss (ARHL) groups over the first six training blocks (slope-I: blocks1-6) and the last seven training blocks (slope-II: blocks 7-13). T-test, and p values of the group comparison are also shown.

		NH	ARHL	t	p	95% confidence interval of the difference
**Speech-in-noise**	Slope-I	-1.90	-1.08	-2.51	0.01	[-1.47, -0.16]
		(0.5)	(0.5)			
	Slope-II	-0.34	-0.30	-0.17	0.86	[-0.53, 0.45]
		(0.2)	(0.7)			
**Time-compressed speech**	Slope-I	-0.05	-0.05			
		(0.05)	(0.06)			
	Slope-II	-0.01	-0.005			
		(0.03)	(0.02)			
**Competing speaker**	Slope-I	-2.24	-1.57	-1.69	0.09	[-1.46, 0.13]
		(0.4)	(0.5)			
	Slope-II	0.20	-0.20	-0.004	0.99	[-0.63, 0.63]
		(0.6)	(0.7)			

Legend to Figure1 supplementary: Learning curves. Mean thresholds as a function of the trained blocks for trained Normal-Hearing (NH) and trained Age-Related Hearing Loss (ARHL) participants in (A) Speech-in-noise (B) Time-compressed speech and (C) Competing speaker conditions. Mean signal-to-noise ratio (SNR) thresholds of each block was used as the dependent measure in speech-in-noise and competing speaker conditions and the compression ratio was used for the time-compressed speech condition. Regression lines and slopes of the learning curves for trained NH are shown in red and for trained ARHL in green. Strait lines indicate mean slopes of blocks 1-6 (slope-I) , dashed lines indicate mean slopes of blocks 7-13 (slope-II). ** $p < 0.01$.
