# Supplementary figures and images for: Auditory Perceptual Learning in Adults with and without Age-Related Hearing Loss
Source: Front Psychol. 2016 Feb 3;6:2066. doi: 10.3389/fpsyg.2015.02066 (PMC4737899; doi:10.3389/fpsyg.2015.02066)

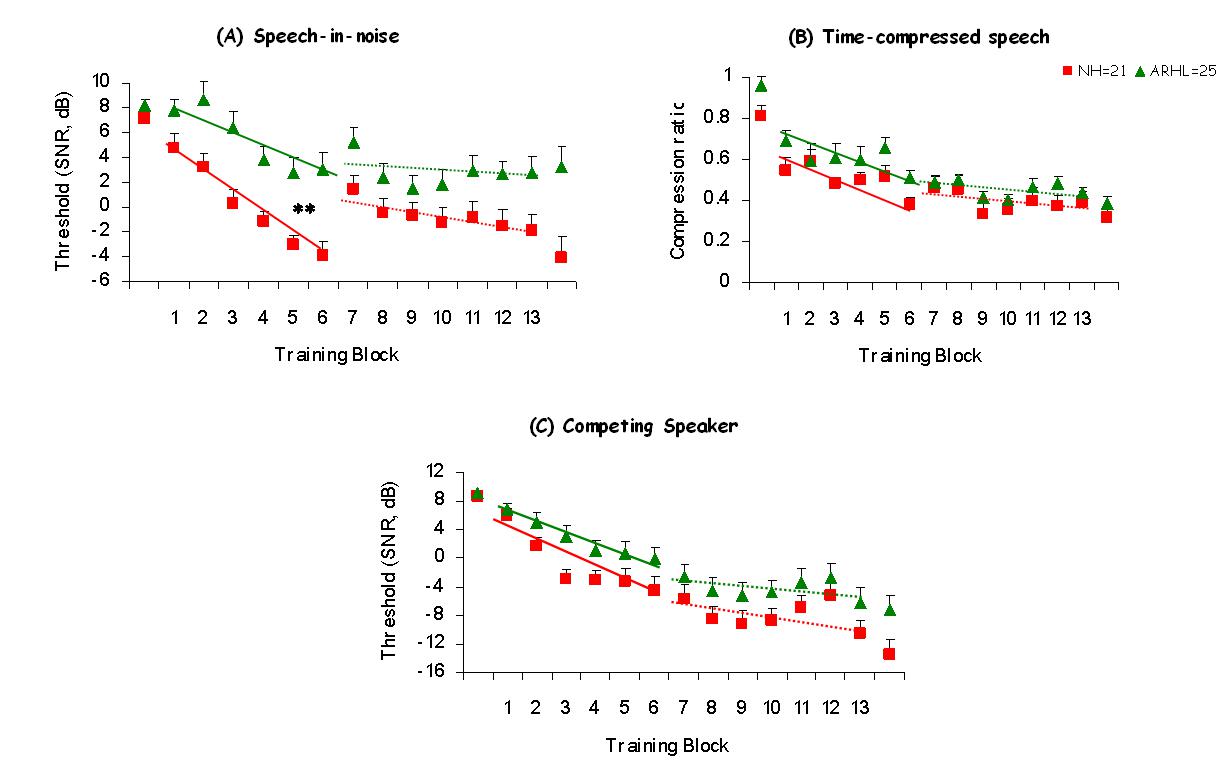

Supplement: Supplementary file 2 [file Image1.JPEG]
